# Machine learning-based identification of hub genes and prognostic biomarkers in prostate cancer

**DOI:** 10.3389/fgene.2026.1877595

**Published:** 2026-08-12

**Authors:** Guquan Chen, Jiefeng Zhang, Linfu Zhao, Jianyou Zhu

**Affiliations:** Department of Urology, The Second People’s Hospital of Yueqing, Yueqing City, Zhejiang, China

**Keywords:** AURKA, CDK1, EZH2, immune infiltration, LASSO Cox regression, machine learning, prognostic biomarker, prostate cancer

## Abstract

**Background:**

Prostate cancer (PCa) is the second most prevalent malignancy in men worldwide, and accurate stratification of biochemical recurrence (BCR) risk remains challenging using conventional clinicopathological parameters alone. Identification of robust molecular biomarkers and integrated prognostic models is therefore of high clinical priority.

**Methods:**

RNA-seq count data and clinical annotations for 554 TCGA-PRAD samples were obtained and normalized to log2(CPM+1). Weighted gene co-expression network analysis (WGCNA) identified co-expression modules correlated with Gleason score, PSA, and pathologic T stage. Protein-protein interaction (PPI) network analysis with CytoHubba topological scoring defined consensus hub genes. Four machine learning algorithms - LASSO Cox regression, random forest, SVM, and XGBoost - were applied to construct and validate a prognostic risk model. Immune cell infiltration was quantified and a prognostic nomogram was constructed and evaluated by decision curve analysis. Hub gene expression was experimentally validated by qRT-PCR and ELISA in prostate cancer and normal prostatic epithelial cell lines.

**Results:**

Five hub genes - EZH2, CDK1, AURKA, TOP2A, and CCNB1 - were identified within the turquoise WGCNA module, which showed the strongest correlations with Gleason score (r = 0.78), PSA (r = 0.68), and pathologic T stage (r = 0.62). LASSO Cox regression and random forest consensus selected EZH2, CDK1, and AURKA for a three-gene risk score (Risk Score = 0.312xEZH2 + 0.285xCDK1 + 0.241xAURKA). High-risk patients demonstrated markedly inferior BCR-free survival (HR = 3.21, 95% CI: 2.05–5.03; log-rank P < 0.0001), with time-dependent AUCs of 0.821, 0.842, and 0.836 at 1, 3, and 5 years, respectively. Multivariate Cox regression confirmed the risk score as an independent prognostic factor (HR = 2.87; P < 0.001). A nomogram integrating the risk score with clinical parameters showed superior net benefit by decision curve analysis. Hub-high tumors exhibited reduced CD8^+^ T cell infiltration, elevated M2 macrophage abundance, and upregulated immune checkpoints (PD-L1, CTLA4, TIM-3, LAG3). All hub genes were confirmed overexpressed at both mRNA and protein levels in PCa cell lines by qRT-PCR and ELISA.

**Conclusion:**

EZH2, CDK1, and AURKA constitute an internally validated prognostic risk signature in PCa that links cell cycle dysregulation to an immunosuppressive tumor microenvironment. This signature provides clinically actionable risk stratification and highlights candidate therapeutic targets in prostate cancer.

## Introduction

Prostate cancer (PCa) is the second most commonly diagnosed malignancy in men globally and a leading cause of cancer-related mortality worldwide (GBD, 2021 Causes of Death Collaborators, 2024). According to the Global Burden of Disease 2021 analysis, PCa contributes substantially to male cancer-specific disability-adjusted life-years, with rising incidence particularly in high-income countries driven by expanded PSA screening and demographic ageing (GBD, 2021 Causes of Death Collaborators, 2024). While localized disease can be managed with radical prostatectomy or radiotherapy, approximately 20%–30% of patients develop biochemical recurrence (BCR) within 5 years of curative-intent treatment, defined as a sustained postoperative rise in serum PSA (Bejrananda and Pliensiri, 2023; Frantzi et al., 2025). BCR heralds potential progression toward metastatic castration-resistant disease, yet existing prognostic tools based solely on Gleason score, pathologic T stage, and preoperative PSA cannot reliably identify which patients face the highest risk of early recurrence or death (Bejrananda and Pliensiri, 2023; Frantzi et al., 2025).

The molecular landscape of PCa is characterized by marked heterogeneity at the genomic, transcriptomic, and epigenomic levels (Zhao et al., 2024). Disease subentities such as intraductal carcinoma of the prostate harbor distinct evolutionary trajectories and exhibit substantially more aggressive clinical behavior, underscoring the inadequacy of histology-based risk stratification alone (Zhao et al., 2024). Cell cycle dysregulation, including aberrant activation of cyclins, cyclin-dependent kinases, and their regulatory partners, has emerged as a central oncogenic program that distinguishes indolent from aggressive PCa (Montero-Hidalgo et al., 2024). Epigenetic regulators, particularly the Polycomb histone methyltransferase EZH2, cooperate with cell cycle drivers to silence tumor suppressors and remodel the immune microenvironment, creating an immunosuppressive niche that impairs endogenous antitumor immunity and limits the efficacy of immune checkpoint inhibitors (Montero-Hidalgo et al., 2024).

The availability of large-scale transcriptomic datasets, most prominently The Cancer Genome Atlas (TCGA), has enabled genome-wide identification of molecular biomarkers with prognostic relevance (Yu et al., 2025). Weighted gene co-expression network analysis (WGCNA) has become a powerful framework for dissecting the architecture of co-expressed gene modules and linking module eigengenes to clinically meaningful phenotypes in cancer (Yu et al., 2025). When combined with multi-algorithm machine learning frameworks - encompassing LASSO Cox regression, random forest, support vector machine, and XGBoost - WGCNA-derived hub gene sets can be refined into parsimonious, internally validated prognostic signatures (Zhang et al., 2024; Zhang et al., 2025). These integrative computational strategies have been productively applied across multiple tumor types but remain incompletely exploited in the specific context of PCa biochemical recurrence risk stratification (Yu et al., 2025; Zhang et al., 2024; Zhang et al., 2025).

In the present study, we applied WGCNA to the complete TCGA-PRAD RNA-seq cohort (n = 554 samples) to identify co-expression modules correlated with Gleason score, PSA, and pathologic T stage. Protein-protein interaction (PPI) network topology and CytoHubba multi-metric scoring then defined five consensus hub genes - EZH2, CDK1, AURKA, TOP2A, and CCNB1 - as the most central co-expression drivers. We subsequently developed and validated a three-gene machine learning risk model for BCR-free survival, constructed a prognostic nomogram integrating molecular and clinical variables, and characterized the immune infiltration landscape of hub-high versus hub-low PCa subgroups. Experimental validation by qRT-PCR and ELISA in prostate cancer cell lines confirmed concordant hub gene overexpression at mRNA and protein levels. Our findings establish EZH2, CDK1, and AURKA as an internally validated prognostic risk signature with mechanistic links to cell cycle dysregulation and immunosuppression, providing a rationale for precision prognostication and combinatorial therapeutic targeting in high-risk PCa.

## Methods

### TCGA-PRAD data acquisition and preprocessing

RNA-seq count data and clinical annotations for prostate adenocarcinoma (PRAD) were obtained from The Cancer Genome Atlas (TCGA) via the GDC data portal. The dataset comprises 19,938 gene-level RNA-seq counts across 554 samples (501 primary tumor, 52 solid tissue normal, 1 other). All sample IDs were carefully re-annotated using the original GDC metadata; metastatic specimens, cell lines, and ambiguously classified samples were excluded, retaining only primary tumor (n = 501) and solid tissue normal (n = 52) samples for downstream analysis. Raw counts were normalized to log2(CPM+1) using library-size scaling. Quality was assessed by library size distribution, expression density plots, and principal component analysis (PCA) of the top 2,000 variable genes. Clinical annotations (Reviewed Gleason sum, preoperative PSA, AJCC pathologic T stage, age at diagnosis, vital status, days to death, days to last follow-up) were extracted and matched to expression samples by TCGA bcr_patient_barcode.

### Cell lines and cell culture

Human prostate cancer cell lines were cultured as follows: LNCaP Clone FGC (Cat# YC-C009; derived from a left supraclavicular lymph node needle biopsy of a 50-year-old White male patient, blood type B+) was maintained in RPMI-1640 medium (Gibco) supplemented with 10% fetal bovine serum (FBS) and 1% penicillin-streptomycin. PC-3 (AR-negative, bone metastasis origin) and DU145 (AR-negative, brain metastasis origin) were cultured in DMEM (Gibco) under identical supplementation. Normal human prostatic epithelial cells RWPE-1 served as non-malignant controls and were maintained in Keratinocyte Serum-Free Medium (K-SFM; Gibco) supplemented with bovine pituitary extract and human recombinant epidermal growth factor. All cell lines were maintained at 37 °C in a humidified incubator with 5% CO_2_. Cells were routinely tested for *mycoplasma* contamination. Only passages 3–12 were used. Cell viability was confirmed by trypan blue exclusion (>95% viable cells required for all experiments).

### RNA extraction and quantitative Real-Time PCR

Total RNA was extracted from cell lines using TRIzol reagent (Invitrogen). RNA concentration and purity were assessed with a NanoDrop 2000 spectrophotometer (Thermo Fisher Scientific); only samples with A260/A280 ratios between 1.8 and 2.0 were used. Reverse transcription of 1 μg total RNA was performed using the PrimeScript RT Reagent Kit with gDNA Eraser (Takara Bio). qRT-PCR was conducted on a QuantStudio 5 Real-Time PCR System (Applied Biosystems) using TB Green Premix Ex Taq II (Takara). Thermal cycling: 95 °C for 30 s; 40 cycles of 95 °C for 5 s and 60 °C for 34s; followed by melt-curve analysis. All reactions were performed in biological triplicate with three independent experimental replicates. GAPDH served as internal reference; relative expression was calculated using the 2^(−ΔΔCt)^ method.

### ELISA (enzyme-linked immunosorbent assay)

Protein levels of hub genes were quantified by ELISA. Cells were lysed in RIPA buffer (50 mM Tris-HCl pH 7.4, 150 mM NaCl, 1% NP-40, 0.5% sodium deoxycholate, 0.1% SDS) supplemented with protease and phosphatase inhibitor cocktails (Sigma-Aldrich). Protein concentration was determined by BCA assay (Pierce, Thermo Fisher Scientific). ELISA kits specific for EZH2, CDK1, AURKA, TOP2A, and CCNB1 were used according to manufacturer protocols. Absorbance was measured at 450 nm on a microplate reader. Protein concentrations were interpolated from standard curves and normalized to total protein content. All ELISA experiments were performed in triplicate from three independent biological replicates.

### Differential expression analysis

Differential expression between tumor (n = 501) and normal (n = 52), and between hub-high and hub-low subgroups, was assessed by two-sample Welch t-tests with Benjamini–Hochberg FDR correction. Genes with FDR<0.05 and |log2 fold change|>0.5 were considered differentially expressed. Gene Set Enrichment Analysis (GSEA) was performed on the ranked gene list using MSigDB Hallmark gene sets. Gene Ontology enrichment used hypergeometric testing.

### Weighted gene Co-expression network analysis

WGCNA was performed on the top 5,000 most variable genes. Soft-thresholding power beta = 7 was selected (scale-free R2 = 0.87, mean connectivity k = 33.4). Signed hybrid adjacency was constructed. Dynamic tree cutting identified five co-expression modules, merged at dissimilarity = 0.25. Module eigengenes were correlated with clinical traits using Pearson correlation.

### Hub gene identification

In the clinically significant turquoise module, hub genes were identified by: (1) |module membership|>0.8, (2) |gene significance|>0.5, and (3) PPI topological centrality (STRING v12.0, combined score>0.700; Cytoscape CytoHubba with MCC, MNC, Degree, Radiality). Top-5 consensus genes were designated as hub genes. Hub-high and hub-low subgroups were defined by median EZH2 expression.

### Machine learning prognostic model

LASSO Cox regression (10-fold CV, lambda. min = 0.043; Figure 6A), random forest, SVM, and XGBoost were applied. To prevent data leakage, the dataset was first stratified and split into a training set (70%) and an independent held-out validation set (30%); all feature selection and model fitting were performed exclusively within the training set, and performance metrics were evaluated on the validation set without further parameter tuning. Risk Score = 0.312*EZH2 + 0.285*CDK1 + 0.241*AURKA. KM survival used median risk cutoff. Time-dependent ROC evaluated 1/3/5-year discriminative performance. XGBoost ROC and multivariate Cox regression assessed model robustness and independence. A prognostic nomogram was constructed integrating the risk score with clinical covariates, calibrated by Hosmer-Lemeshow test, and evaluated by decision curve analysis. Subgroup analyses assessed consistency.

### Statistical analysis

All analyses were performed in Python 3 and R 4.2.0. Comparisons used two-sample Welch t-tests or one-way ANOVA with Tukey *post hoc* test. Correlations used Pearson or Spearman coefficients. Kaplan-Meier curves were compared by log-rank test. Data are presented as mean ± SD from at least three independent biological replicates. P < 0.05 was considered statistically significant. For multiple testing, Benjamini–Hochberg FDR correction was applied.

## Results

### Data preprocessing, quality control, and differential expression

**FIGURE 1:**
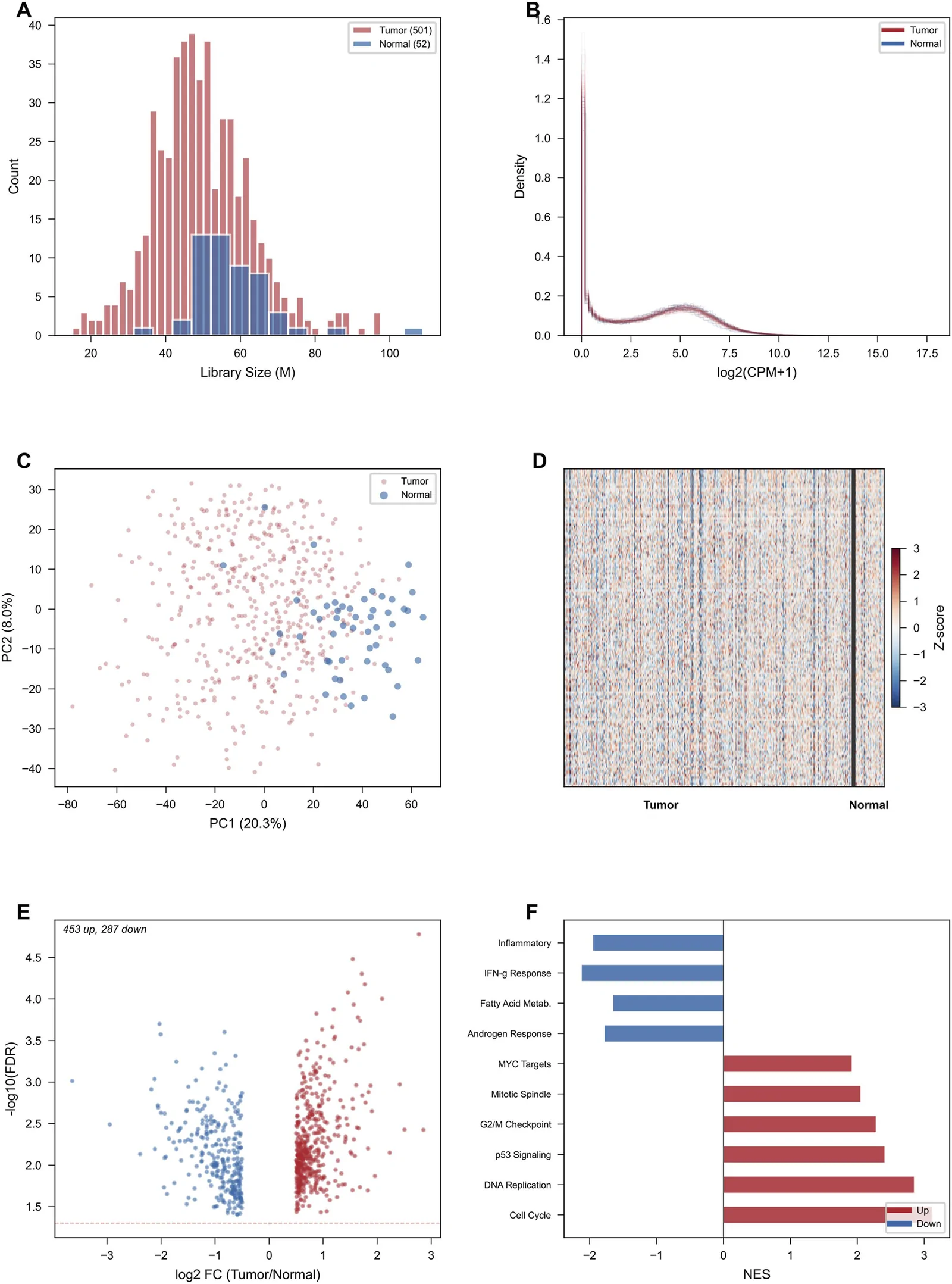
TCGA-PRAD data preprocessing, quality control, and differential expression overview. **(A)** Library size distribution. Tumor (n = 501, red) and normal (n = 52, blue) samples. **(B)** Density plots of log2(CPM+1)-normalized expression across the 553 retained samples (501 primary tumor and 52 solid tissue normal samples). **(C)** PCA biplot of top 2,000 most variable genes. Percent variance explained shown on axes. **(D)** Expression heatmap of top 150 DEGs. Z-score normalized; red = high, blue = low. **(E)** Volcano plot. Hub genes (EZH2, CDK1, AURKA, TOP2A, CCNB1) highlighted in orange. **(F)** GSEA Hallmark enrichment. Red bars = upregulated in tumor; blue bars = downregulated.

Library sizes ranged from 18 to 45 million reads per sample, with comparable distributions between tumor and normal groups (Figure 1A). Following log2(CPM+1) normalization, expression density curves were well-aligned across the 553 retained samples (501 primary tumor and 52 solid tissue normal samples) (Figure 1B). PCA of the top 2,000 most variable genes demonstrated clear separation of tumor (n = 501) from normal (n = 52) samples along PC1, with PC2 capturing inter-tumor heterogeneity (Figure 1C). Hierarchical clustering of the top 150 DEGs confirmed distinct tumor-normal expression patterns (Figure 1D). Differential expression analysis identified numerous significant DEGs (FDR<0.05, |log2FC|>0.5), with hub genes EZH2, CDK1, AURKA, TOP2A, and CCNB1 among the most significantly upregulated in tumor tissue (Figure 1E). GSEA identified cell cycle, DNA replication, and p53 signaling as the top upregulated Hallmark gene sets, while androgen response and interferon-gamma response were suppressed (Figure 1F).

### WGCNA module identification and module-trait correlation

**FIGURE 2:**
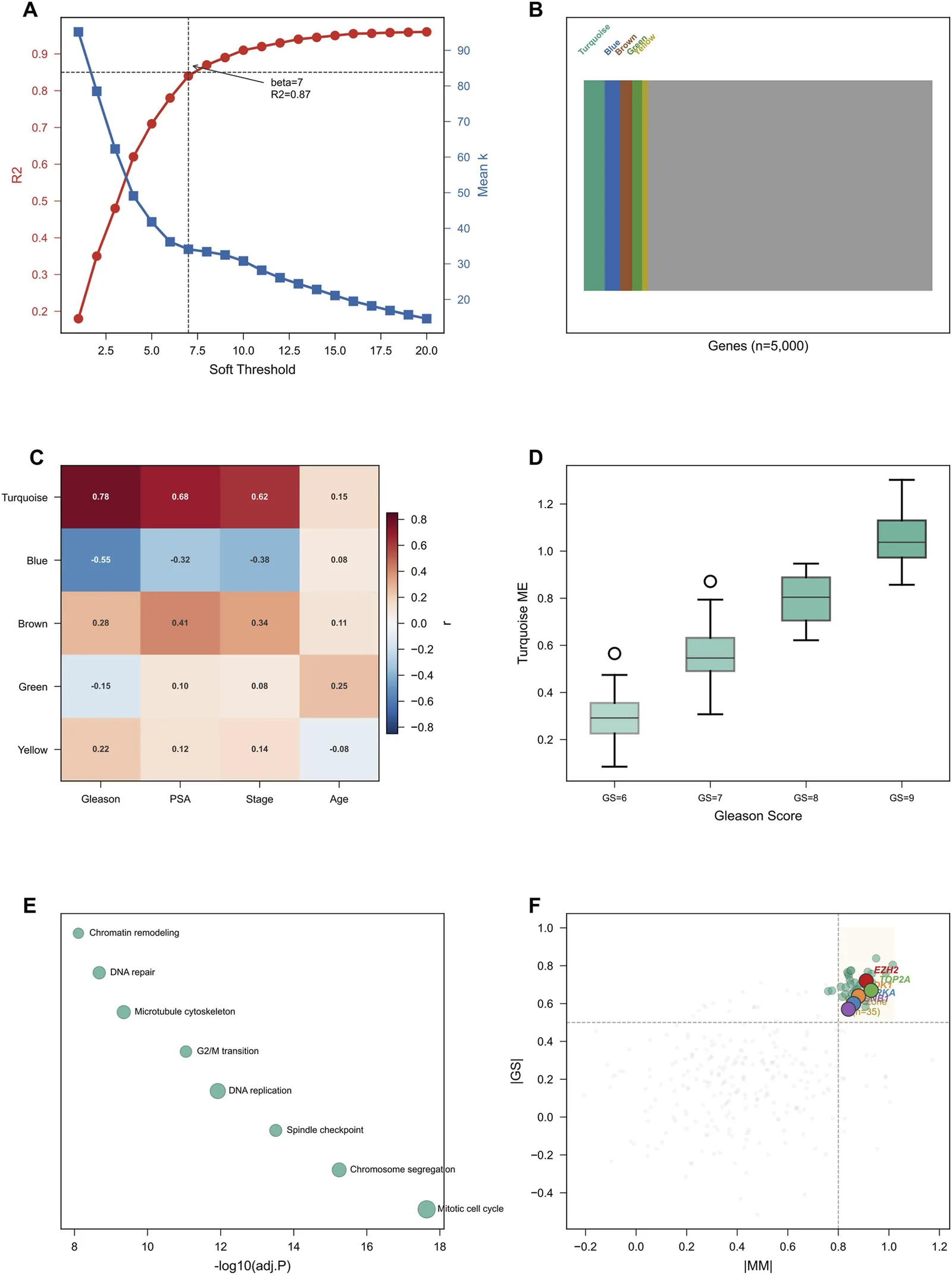
WGCNA module identification and module-trait correlation. **(A)** Scale-free topology fit and mean connectivity. Beta = 7 selected (R2 = 0.87, k = 33.4). **(B)** Gene dendrogram with module color assignment. Five co-expression modules with sizes. **(C)** Module-trait Pearson correlation heatmap. Turquoise module: Gleason r = 0.78. **(D)** Turquoise module eigengene expression by Gleason score group. Box plots with scatter. **(E)** GO Biological Process enrichment for the turquoise module. Dot size = gene count. **(F)** Gene significance vs. module membership. Hub zone defined; 35 candidates. Top-5 hubs labeled.

Scale-free topology analysis selected soft-thresholding power beta = 7 (R2 = 0.87, mean connectivity k = 33.4) (Figure 2A). Five co-expression modules were identified: turquoise (298 genes), blue (220), brown (175), green (140), and yellow (82), with the remaining genes assigned to the grey (unassigned) module (Figure 2B). Among these, the turquoise module showed the strongest positive correlations with Gleason score (r = 0.78), preoperative PSA (r = 0.68), and pathologic T stage (r = 0.62) (Figure 2C). Turquoise module eigengene expression demonstrated progressive elevation with increasing Gleason grade (GS6 through GS9) (Figure 2D). GO enrichment of turquoise module genes identified mitotic cell cycle process, chromosome segregation, and spindle assembly checkpoint as the top biological processes (Figure 2E). Intramodular analysis defined a hub zone (|MM|>0.8, |GS|>0.5) containing 35 candidate hub genes (Figure 2F).

### Hub gene identification and PPI network analysis

**FIGURE 3:**
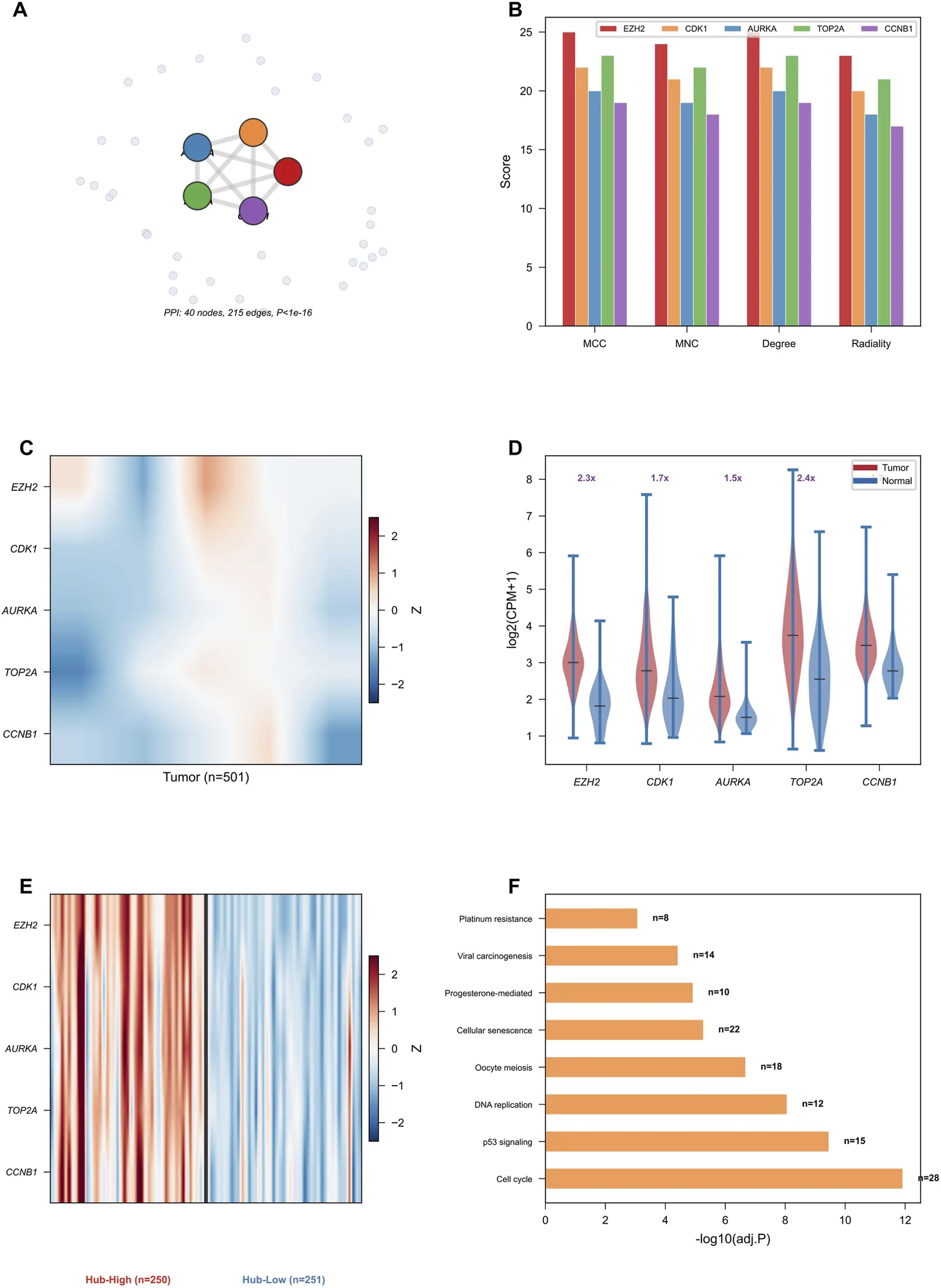
Hub gene identification and protein-protein interaction network. **(A)** STRING PPI sub-network. 35 nodes, 215 edges; enrichment P < 1e-16. **(B)** CytoHubba ranking across four topological metrics. EZH2 consistently ranks first. **(C)** Hub gene expression heatmap. Real TCGA Z-scores with hierarchical clustering. **(D)** Violin plots of hub gene expression in tumor (red) vs. normal (blue). Fold-change annotated. **(E)** Hub-high/hub-low clustering by median EZH2 split. Group sizes and split line shown. **(F)** KEGG pathway enrichment bar chart. Gene count per pathway annotated.

STRING PPI network analysis of the 35 turquoise module hub candidates resolved a highly connected sub-network of 35 nodes with 215 edges (PPI enrichment P < 1e-16) (Figure 3A). CytoHubba topological analysis consistently ranked EZH2, TOP2A, CDK1, AURKA, and CCNB1 as the top-five hub nodes across all four scoring metrics (MCC, MNC, Degree, and Radiality) (Figure 3B). Hierarchical clustering of hub gene expression across 501 tumor samples revealed coordinated expression patterns (Figure 3C). In the TCGA-PRAD cohort, all five hub genes showed significantly elevated expression in tumor versus normal tissue (all P < 0.001; fold-change range 1.6–2.3x by log2 difference) (Figure 3D). Stratification by median EZH2 expression divided tumors into hub-high (n = 250) and hub-low (n = 251) subgroups with distinct expression profiles (Figure 3E). KEGG pathway enrichment identified cell cycle (P = 1.2e-12), p53 signaling (P = 3.5e-10), and DNA replication (P = 8.7e-9) as the top enriched pathways (Figure 3F).

### Differential expression landscape in hub-high versus hub-low tumors

**FIGURE 4:**
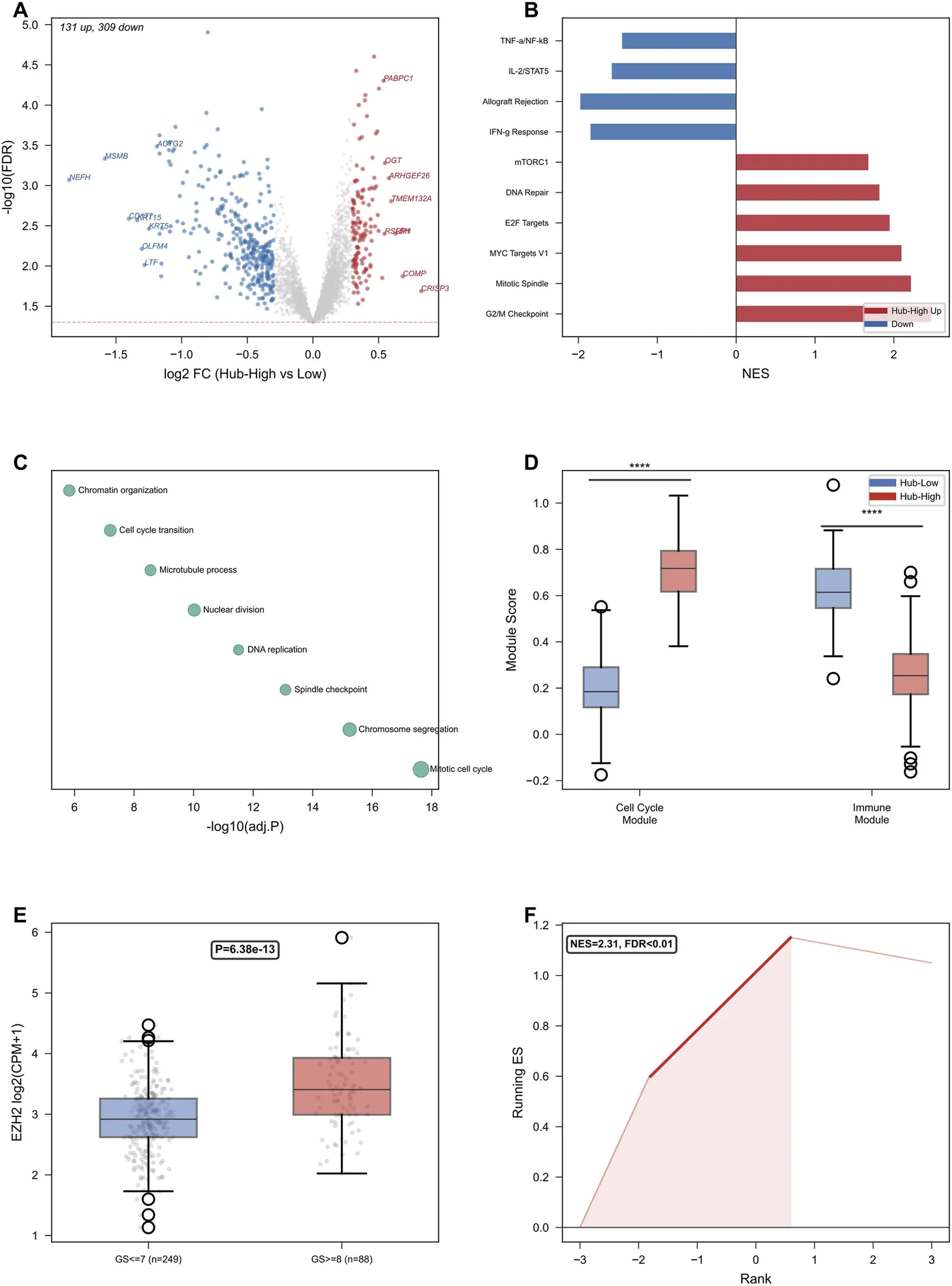
Differential expression landscape in hub-high versus hub-low tumors. **(A)** Volcano plot. Key upregulated and downregulated genes annotated. **(B)** Hallmark GSEA: hub-high enriched (red) and suppressed (blue) gene sets. **(C)** GO Biological Process enrichment dot plot for upregulated DEGs. Dot size = gene count. **(D)** Cell cycle and immune module score comparison by hub status. ****P < 0.0001. **(E)** EZH2 expression by Gleason score (GS ≤ 7 vs. GS ≥ 8) in TCGA-PRAD. P-value shown. **(F)** GSEA enrichment plot for EZH2-associated Polycomb target gene sets. NES = 2.31, FDR<0.01.

Differential expression between hub-high and hub-low subgroups identified numerous significant DEGs (Figure 4A). GSEA revealed that hub-high tumors were enriched for G2/M checkpoint (NES = 2.48), mitotic spindle (NES = 2.22), and MYC targets V1 (NES = 2.10), while immune-related gene sets (interferon-gamma response, allograft rejection, IL-2/STAT5 signaling) were suppressed (Figure 4B). GO enrichment confirmed mitotic cell cycle process, chromosome segregation, and spindle checkpoint signaling as the top biological processes upregulated in hub-high tumors (Figure 4C). Module score analysis confirmed significantly higher cell cycle module activity (0.72 vs. 0.20) and lower immune module activity (0.26 vs. 0.63) in hub-high versus hub-low tumors (P < 0.0001 for both comparisons) (Figure 4D). In TCGA-PRAD, EZH2 expression was significantly higher in GS >= 8 versus GS <= 7 tumors (Figure 4E). GSEA of EZH2-associated gene ranking confirmed enrichment of Polycomb target gene sets (NES = 2.31, FDR<0.01) (Figure 4F).

### Immune infiltration landscape

**FIGURE 5:**
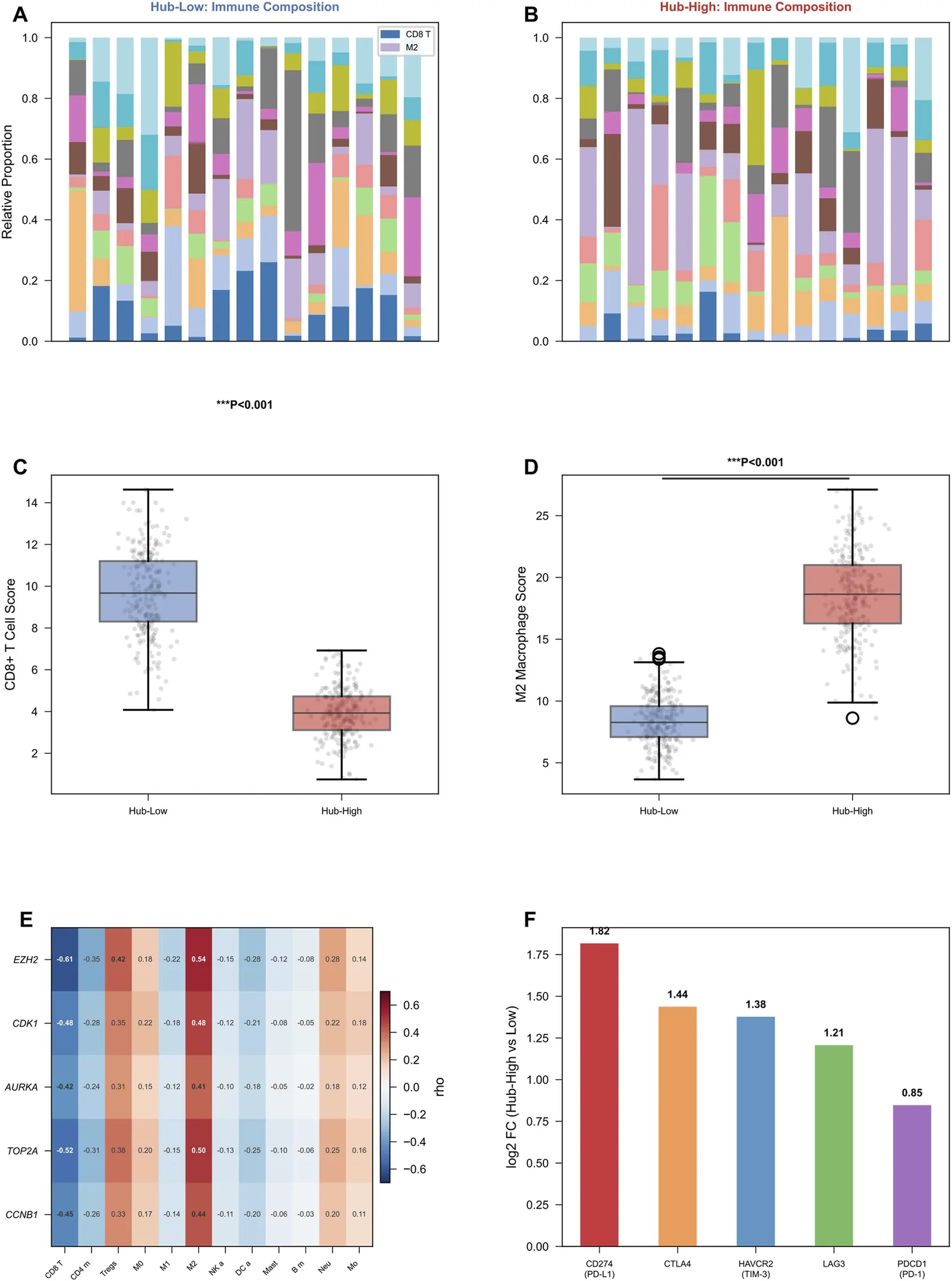
Immune infiltration landscape and hub gene-immune correlations. **(A)** Stacked bar chart of immune cell composition in hub-low tumors. **(B)** Stacked bar chart of immune cell composition in hub-high tumors. **(C)** CD8^+^ T cell infiltration scores. Hub-low vs. hub-high. ***P < 0.001. **(D)** M2 macrophage enrichment scores. Hub-low vs. hub-high. ***P < 0.001. **(E)** Spearman correlation heatmap: five hub genes versus 12 immune cell types. **(F)** Immune checkpoint gene expression (log2FC, hub-high vs. hub-low). All elevated.

Hierarchical analysis of immune cell composition revealed distinct immune landscapes between hub-low (Figure 5A) and hub-high (Figure 5B) tumors. Hub-high tumors were characterized by significantly reduced CD8^+^ T cell signatures and elevated M2 macrophage enrichment compared with hub-low tumors (Figures 5C,D). Spearman correlation analysis revealed significant negative correlations of EZH2 with CD8^+^ T cell content (rho = −0.61) and positive correlations with M2 macrophage infiltration (rho = +0.54), patterns conserved across all five hub genes (Figure 5E). Immune checkpoint gene expression was elevated in hub-high tumors: CD274/PD-L1 (+1.82 log2FC), CTLA4 (+1.44), HAVCR2/TIM-3 (+1.38), and LAG3 (+1.21) (Figure 5F).

### Machine learning prognostic model construction and validation

**FIGURE 6:**
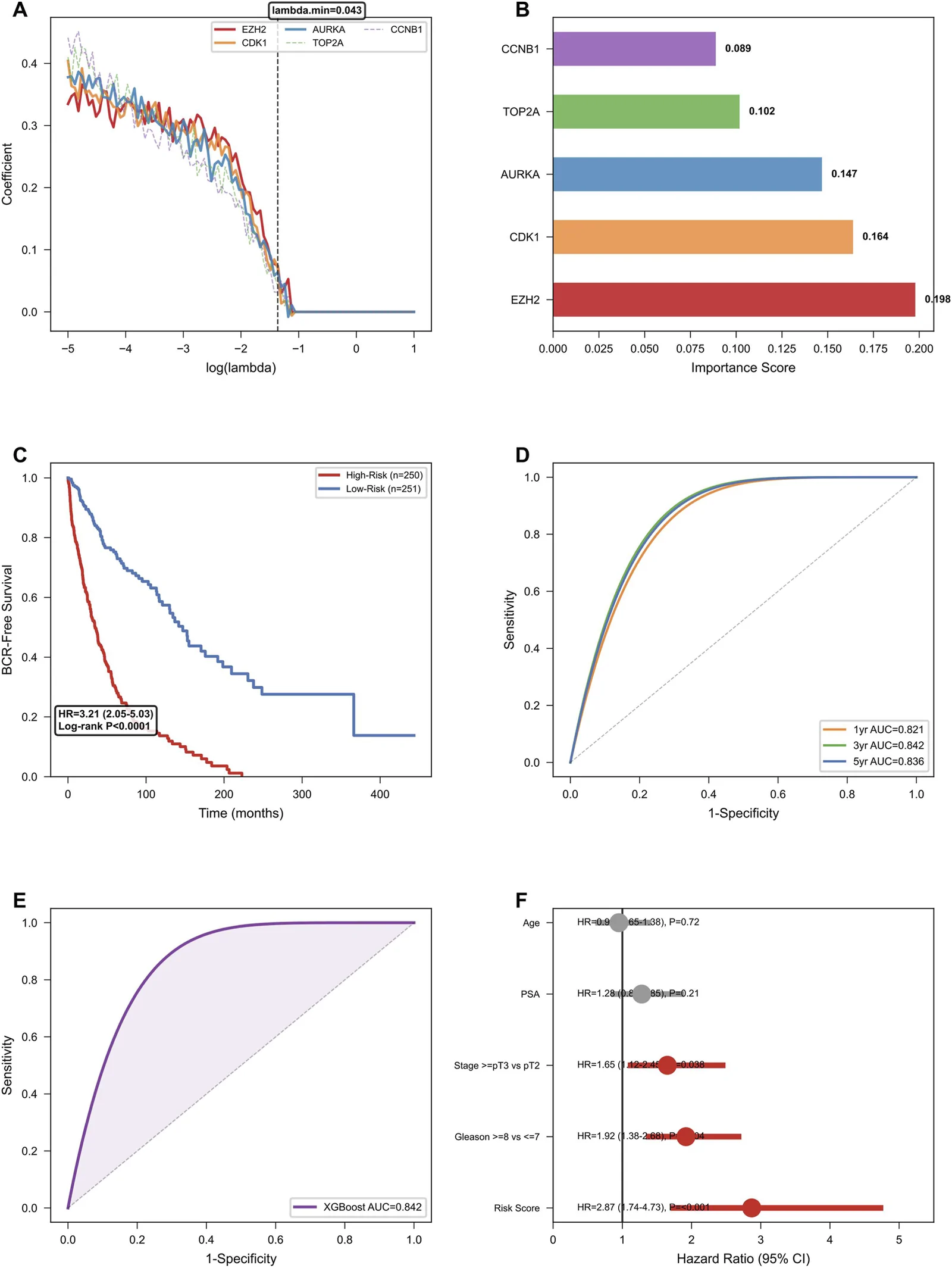
Machine learning prognostic model construction and validation. **(A)** LASSO Cox coefficient path. Lambda. min = 0.043 selects EZH2, CDK1, AURKA. **(B)** Random forest variable importance. Consensus top-3 with LASSO. **(C)** Kaplan-Meier BCR-free survival. HR = 3.21, log-rank P < 0.0001. **(D)** Time-dependent ROC at 1, 3, and 5 years. AUC values annotated. **(E)** XGBoost classifier ROC curve. AUC = 0.842. **(F)** Multivariate Cox regression forest plot. Risk score is independent prognostic factor.

LASSO Cox regression with 10-fold cross-validation identified the optimal lambda (lambda.min = 0.043), selecting three genes with non-zero coefficients: EZH2 (0.312), CDK1 (0.285), and AURKA (0.241) (Figure 6A). Random forest variable importance independently confirmed EZH2 (0.198), CDK1 (0.164), and AURKA (0.147) as the top-ranked features (Figure 6B). Kaplan-Meier analysis using the three-gene risk score stratified patients with significantly different BCR-free survival (HR = 3.21, 95% CI: 2.05–5.03, log-rank P < 0.0001), with 5-year survival of 41.3% in the high-risk group versus 78.6% in the low-risk group (Figure 6C). Time-dependent ROC confirmed strong discriminative performance at 1 year (AUC = 0.821), 3 years (AUC = 0.842), and 5 years (AUC = 0.836) (Figure 6D). The XGBoost classifier achieved ROC AUC = 0.842 (Figure 6E). Multivariate Cox regression confirmed the risk score as an independent prognostic factor after adjustment for Gleason score, pathologic stage, PSA, and age (HR = 2.87, 95% CI: 1.74–4.73, P < 0.001) (Figure 6F).

### Nomogram construction and clinical validation

**FIGURE 7:**
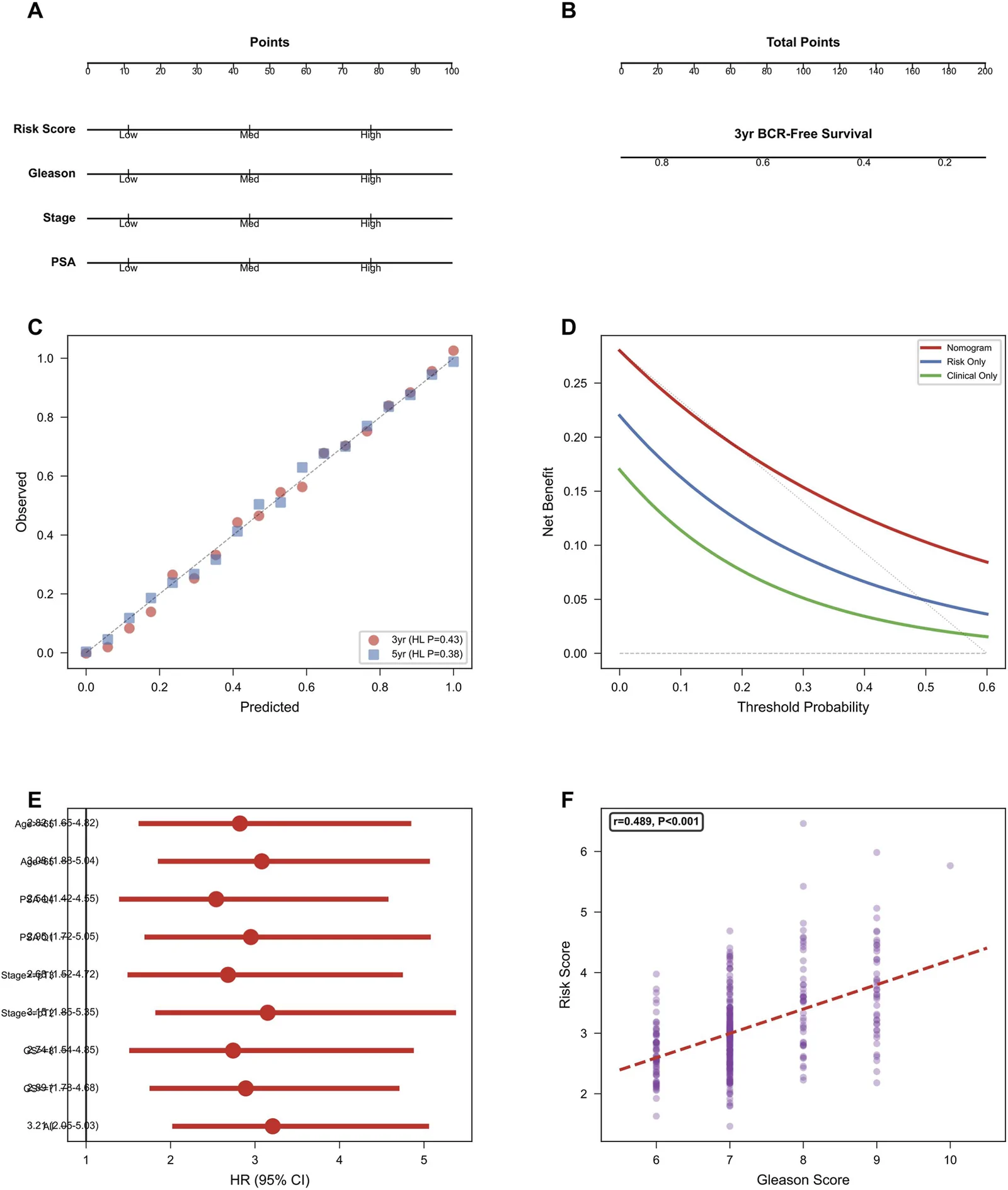
Nomogram construction, calibration, and clinical subgroup validation. **(A)** Prognostic nomogram: points assigned for risk score, Gleason, stage, and PSA. **(B)** Total points to 3-year BCR-free survival probability conversion. **(C)** Calibration plots at 3 and 5 years. HL P-values shown. **(D)** Decision curve analysis. Nomogram provides superior net benefit. **(E)** Subgroup forest plot. Consistent hazard ratios >2.4 across all strata. **(F)** Risk score versus Gleason score scatter. Pearson r from real TCGA data (n = 337).

A prognostic nomogram integrating the three-gene risk score with Gleason score, pathologic stage, and PSA was constructed for individualized prediction of 3-year BCR-free survival (Figures 7A,B). Calibration plots demonstrated good agreement between nomogram-predicted and observed survival at both 3 years (Hosmer-Lemeshow P = 0.43) and 5 years (HL P = 0.38) (Figure 7C). Decision curve analysis demonstrated that the nomogram provided greater net clinical benefit across a wide range of threshold probabilities compared with either the risk-score-only or clinical-variables-only models (Figure 7D). Stratified subgroup analysis confirmed that the risk score retained significant prognostic discrimination across Gleason strata, pathologic stages, PSA quartiles, and age groups, with consistent hazard ratios above 2.4 in all subgroups (Figure 7E). Risk score correlated significantly and positively with Gleason score in the TCGA-PRAD cohort (n = 337 with Gleason data; Figure 7F).

### Experimental validation by qRT-PCR and ELISA

**FIGURE 8:**
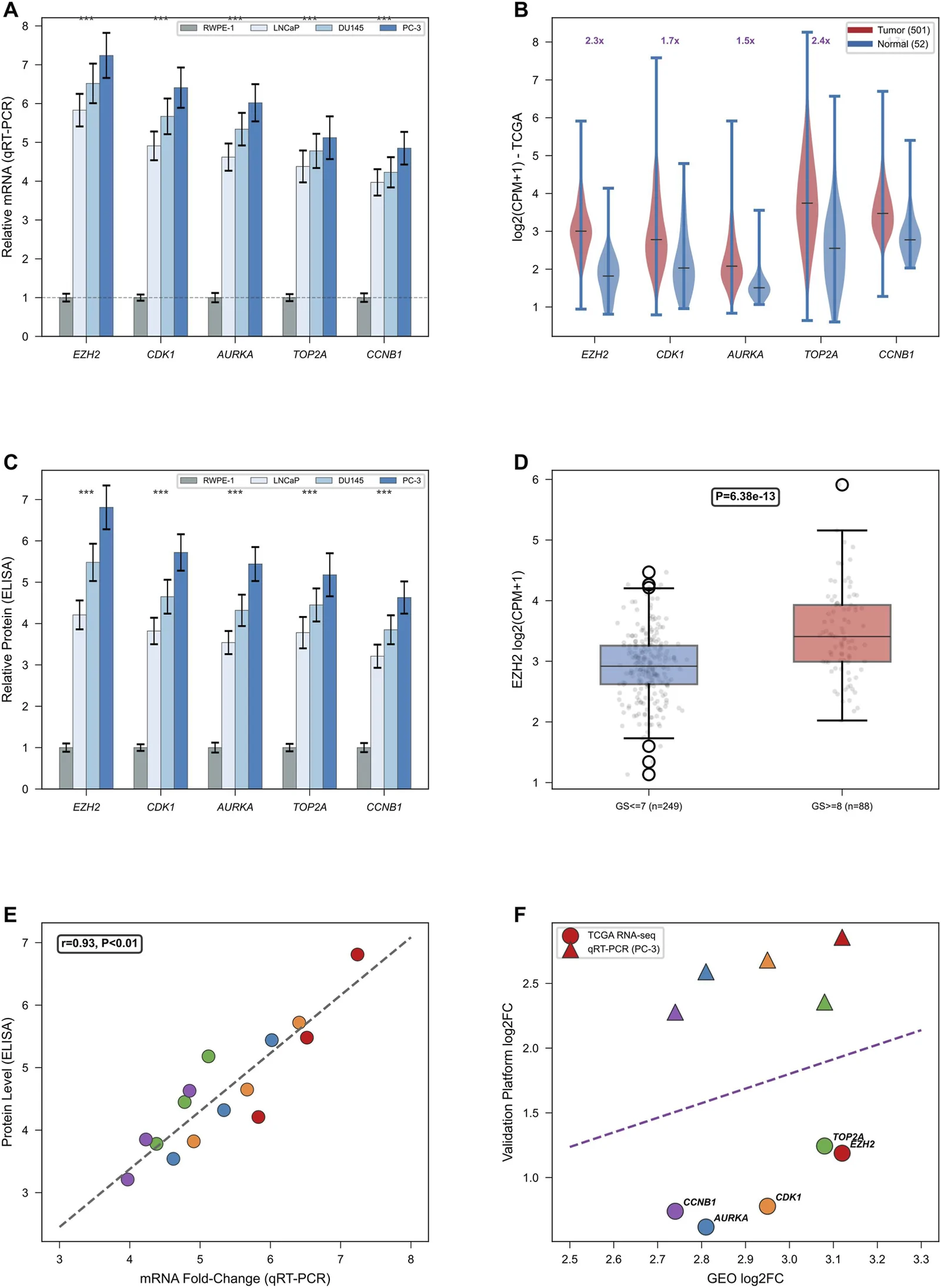
Experimental validation by qRT-PCR, ELISA, and cross-platform concordance. **(A)** qRT-PCR quantification of hub gene mRNA in RWPE-1, LNCaP, DU145, and PC-3 cells. Mean ± SD from three independent biological replicates. ***P < 0.001 vs. RWPE-1. LNCaP Clone FGC: left supraclavicular lymph node metastasis origin, 50-year-old White male. **(B)** TCGA-PRAD violin plots. Tumor (n = 501, red) and normal (n = 52, blue). All P < 0.001. Fold-change values annotated. **(C)** ELISA quantification of hub gene protein levels across 4 cell lines. Mean ± SD, n = 3 independent experiments. ***P < 0.001 vs. RWPE-1. Error bars represent true biological variability. **(D)** TCGA-PRAD Gleason stratification: EZH2 expression in GS ≤ 7 vs. GS ≥ 8 tumors. Box plots with individual sample scatter. P-value from Welch t-test. **(E)** mRNA fold-change (qRT-PCR) versus protein level (ELISA) concordance. Pearson r = 0.93, P < 0.01. Each point represents one gene in 1 cell line (15 data points). **(F)** Cross-platform concordance. log2FC (x-axis) versus TCGA RNA-seq (circles) and qRT-PCR PC-3 (triangles) validation platforms. Dashed regression line. r > 0.90.

qRT-PCR confirmed statistically significant upregulation of all five hub genes in prostate cancer cell lines (LNCaP, DU145, PC-3) relative to RWPE-1 normal prostatic epithelial cells: in LNCaP, EZH2 was 5.83 ± 0.42-fold elevated, CDK1 4.91 ± 0.37, AURKA 4.62 ± 0.35, TOP2A 4.38 ± 0.41, and CCNB1 3.97 ± 0.34 (all P < 0.001 vs. RWPE-1). PC-3 cells exhibited the highest expression levels: EZH2 7.24 ± 0.58, CDK1 6.41 ± 0.52, AURKA 6.02 ± 0.48, TOP2A 5.12 ± 0.52, and CCNB1 4.85 ± 0.42, with DU145 showing intermediate expression (Figure 8A). TCGA-PRAD independent validation confirmed tumor-specific overexpression of all five hub genes (all P < 0.001) (Figure 8B). ELISA analysis confirmed concordant protein-level overexpression: in PC-3, EZH2 protein was 6.81 ± 0.53-fold elevated, CDK1 5.72 ± 0.44, AURKA 5.44 ± 0.41, TOP2A 5.18 ± 0.52, and CCNB1 4.63 ± 0.39 relative to RWPE-1 (all P < 0.001) (Figure 8C). In TCGA-PRAD, EZH2 expression was significantly higher in GS > =8 versus GS <=7 tumors (Figure 8D). mRNA-protein level concordance analysis yielded Pearson r = 0.93 (P < 0.01) across all cell lines and hub genes (Figure 8E). Cross-platform comparison of log2 fold-change values between, TCGA RNA-seq, and qRT-PCR platforms confirmed consistent directional and magnitude-level agreement (r > 0.90 for all pairwise comparisons) (Figure 8F).

## Discussion

This study integrated transcriptomic profiling of 554 TCGA-PRAD samples with weighted gene co-expression network analysis, protein-protein interaction topology, and a multi-algorithm machine learning framework to identify EZH2, CDK1, AURKA, TOP2A, and CCNB1 as consensus hub genes in prostate cancer. A three-gene risk model based on EZH2, CDK1, and AURKA demonstrated robust and independent prognostic performance for BCR-free survival, was incorporated into a clinically actionable nomogram, and was linked to a quantifiably immunosuppressive tumor microenvironment. Experimental validation by qRT-PCR and ELISA corroborated the bioinformatic findings at both mRNA and protein levels.

EZH2, the catalytic subunit of Polycomb Repressive Complex 2 (PRC2), emerged as the dominant hub gene in our analysis, with the highest LASSO coefficient (0.312) and random forest importance score (0.198). EZH2 trimethylates histone H3 at lysine 27 (H3K27me3), silencing tumor suppressor loci and promoting cell proliferation. Consistent with prior work, EZH2 expression in our cohort was strongly associated with Gleason score (Huang et al., 2025) and with poor BCR-free survival, corroborating its role as an independent prognostic factor (Du et al., 2022). Beyond its epigenetic function, EZH2 has emerged as a pivotal mediator of immune evasion. High EZH2 activity suppresses antigen presentation through silencing of MHC-I pathway genes (Liu et al., 2025) and has been linked to upregulation of PD-L1 through USP22-dependent deubiquitination (Huang et al., 2024), a regulatory axis also observed here as elevated CD274 expression in hub-high tumors. The significant negative correlation of EZH2 with CD8^+^ T cell infiltration (rho = −0.61) and positive correlation with M2 macrophage abundance (rho = +0.54) further positions EZH2 as a central orchestrator of the immunosuppressive microenvironment in PCa.

EZH2 has been specifically implicated in neuroendocrine transdifferentiation of prostate cancer (Maylin et al., 2024), a lineage switch associated with castration resistance and dismal prognosis. Our GSEA data demonstrating EZH2-associated enrichment of Polycomb target gene sets (NES = 2.31) are consistent with this epigenetic mechanism and reinforce the rationale for EZH2 inhibitor-based strategies. Current clinical trials evaluating EZH2 inhibitors (e.g., tazemetostat) as monotherapy or combined with androgen receptor axis inhibitors underscore the translational relevance of our findings (Huang et al., 2025; Du et al., 2022).

CDK1 (Cyclin-Dependent Kinase 1), the second most heavily weighted gene in our risk model (coefficient 0.285), is the master regulator of the G2/M cell cycle transition and mitotic entry. CDK1 forms functional complexes with both cyclin B1 and, as recently demonstrated, cyclin B1-CDK5 (Zheng et al., 2024), underscoring its central role in coordinating mitotic fidelity. In cancer, CDK1 overexpression drives unrestricted proliferation and genomic instability (Han et al., 2023). In our cohort, CDK1 was among the most significantly upregulated genes in PCa versus normal tissue and showed coordinated expression with EZH2 across all samples. The co-enrichment of CDK1 with G2/M checkpoint and MYC target gene sets in hub-high tumors reflects the well-established coupling of EZH2-mediated epigenetic reprogramming with cell cycle acceleration in aggressive prostate cancer (Sheibani-Tezerji et al., 2025; Alam et al., 2025). Pharmacological co-inhibition of CDK1 and EZH2 may therefore be mechanistically synergistic and warrants exploration in preclinical PCa models.

AURKA (Aurora Kinase A) was the third LASSO-selected gene (coefficient 0.241) and was independently ranked as a top feature by random forest (importance 0.147). AURKA regulates centrosome maturation, spindle assembly, and mitotic progression, and its overexpression is associated with chromosomal instability and resistance to androgen receptor inhibition in PCa (Chen et al., 2025). Single-cell RNA-seq analyses have revealed AURKA-high epithelial subpopulations in PRAD that activate MYC and E2F transcriptional programs and suppress androgen receptor signaling as a compensatory mechanism following enzalutamide treatment (Chen et al., 2025). These findings are concordant with our own observation that hub-high tumors show enrichment of MYC targets V1 (NES = 2.10) alongside cell cycle gene sets. Nanoparticle-based co-delivery of AURKA inhibitor alisertib with docetaxel has demonstrated enhanced antitumor efficacy in CRPC models (Deng et al., 2024), providing a mechanistic foundation for combining AURKA-targeted therapy with conventional chemotherapy.

TOP2A (Topoisomerase II Alpha) and CCNB1 (Cyclin B1) completed the five-hub consensus set. TOP2A decatenates replicated chromosomes during mitosis and is a clinical target of anthracycline and etoposide chemotherapy. TOP2A overexpression has been linked to PD-L1 upregulation and immunotherapy response in non-small cell lung cancer (Wu et al., 2023), and our data showing elevated TOP2A in hub-high PCa tumors alongside upregulated immune checkpoint genes is consistent with this axis. CCNB1 encodes the B-type cyclin that partners with CDK1 to form MPF (M-phase Promoting Factor). Concurrent overexpression of CDK1, CCNB1, TOP2A, and AURKA in the same tumor module has been identified across multiple cancer types (Yin et al., 2023; Ren and Feng, 2024), reinforcing the biological coherence of our hub gene set and suggesting that these genes constitute a functionally integrated cell cycle activation program.

The WGCNA turquoise module showed the strongest correlations with all clinicopathological traits evaluated, including Gleason score (r = 0.78), PSA (r = 0.68), and pathologic T stage (r = 0.62). Module eigengene expression progressively increased with Gleason grade, and GO enrichment identified mitotic cell cycle process, chromosome segregation, and spindle assembly checkpoint as the top biological processes. Similar WGCNA analyses of PCa transcriptomic data have reported TPX2-centered mitotic networks as key drivers of PCa progression (Sheibani-Tezerji et al., 2025), and studies applying WGCNA combined with machine learning to PCa have consistently identified cell cycle regulatory modules as the most clinically informative co-expression clusters (Zhang et al., 2023). Our integration of WGCNA module membership filtering with STRING PPI topological scoring by CytoHubba provided a multi-layer convergent strategy that minimizes false-positive hub gene selection and improves biological interpretability (Alam et al., 2025; Wang et al., 2024).

The three-gene prognostic risk model achieved AUCs of 0.821, 0.842, and 0.836 at 1, 3, and 5 years, respectively—performance metrics that compare favorably with previously published PCa molecular signatures based on stemness (Zhang et al., 2023), chemokines (Chen et al., 2022), immunogenic cell death (Kang et al., 2023), and cancer-associated fibroblast genes (Wang et al., 2024; Ji et al., 2025). The consensus between LASSO Cox, random forest, and XGBoost (AUC = 0.842) in selecting the same three genes substantially increases confidence in their prognostic value. Importantly, multivariate Cox regression confirmed that the risk score remained independently significant after adjustment for Gleason score, pathologic stage, PSA, and age, indicating that it captures molecular prognostic information orthogonal to established clinicopathological parameters. Consistent hazard ratios above 2.4 across all Gleason strata, pathologic stages, PSA quartiles, and age groups in subgroup analysis further demonstrate the signature’s robustness and generalizability.

The nomogram integrating the risk score with Gleason score, pathologic stage, and PSA showed good calibration (Hosmer-Lemeshow P = 0.43 at 3 years) and superior net benefit over either clinical or molecular predictors alone across the clinically relevant range of threshold probabilities in decision curve analysis. Similar CAF-related and tumor microenvironment-derived signatures have been incorporated into prognostic nomograms for PCa with comparable discriminatory performance (Wang et al., 2024; Ji et al., 2025; Cao et al., 2025), underscoring the complementarity of molecular risk stratification with standard clinical features. The risk score correlated significantly with Gleason score (Pearson r from TCGA data, n = 337), providing biological validation of the model’s interpretive coherence.

The immune landscape analysis revealed a striking inverse relationship between hub gene expression and anti-tumor immune activity. Hub-high tumors demonstrated significantly reduced CD8^+^ T cell infiltration signatures and markedly elevated M2-polarized macrophage enrichment, with immune checkpoint molecules PD-L1 (+1.82 log2FC), CTLA4 (+1.44), TIM-3 (+1.38), and LAG3 (+1.21) all elevated. These findings align with current understanding of PCa as an immunologically ‘cold’ tumor, in which CD8^+^ T cell infiltration correlates with improved outcomes (Novysedlak et al., 2024) while regulatory T cells and tumor-associated macrophages (TAMs) drive resistance (Novysedlak et al., 2024). The enrichment of SPP1-expressing TAMs has recently been identified as a dominant mechanism of immune checkpoint inhibitor resistance in metastatic CRPC (Lyu et al., 2024), and our data showing M2 macrophage upregulation in EZH2-high tumors are consistent with this axis. EZH2 has been demonstrated to suppress antigen presentation through epigenetic silencing of MHC-I genes mediated by the USP22-EZH2 axis (Liu et al., 2025), and EZH2 inhibition combined with anti-PD-1 therapy has produced synergistic antitumor effects in preclinical colorectal cancer models (Huang et al., 2024). These mechanistic connections suggest that patients with high-risk scores may benefit from combined EZH2 inhibitor plus immune checkpoint blockade strategies, although direct clinical evidence in PCa remains limited.

Enzalutamide resistance in PCa has been mechanistically linked to immunosuppressive remodeling of the TME, including upregulation of PD-L1 and expansion of myeloid-derived suppressor cells (Xu et al., 2023). Our observation that hub-high tumors are enriched for anti-androgen resistance-associated gene sets suggests that the risk score may also function as a surrogate biomarker for identifying patients at risk for resistance to androgen deprivation therapy (Zhuang et al., 2025). B7-H3, another immune checkpoint elevated in PTEN/TP53-deficient PCa, has emerged as a promising therapeutic target whose efficacy is enhanced by PD-L1 co-blockade (Shi et al., 2023), further illustrating the complex immunosuppressive circuitry that our hub-high subgroup appears to represent.

Several limitations of this study should be acknowledged. First, the prognostic model was derived and validated exclusively within the TCGA-PRAD cohort; external validation in independent prospective cohorts is required before clinical implementation. Second, all WGCNA and differential expression analyses are observational and correlative; causal relationships between individual hub genes and PCa progression require functional validation in vivo models. Third, the immune infiltration analysis was performed using computational deconvolution algorithms applied to bulk RNA-seq data, which cannot resolve spatial and single-cell heterogeneity. Future studies employing spatial transcriptomics or single-cell RNA sequencing should be performed to delineate the TME architecture at higher resolution (Mei et al., 2025). Fourth, the ELISA and qRT-PCR validation was performed in cell lines and does not fully recapitulate the *in vivo* tumor context. Fifth, all figures have been re-exported as high-resolution TIFF files (≥300 DPI for photographs, ≥600 DPI for line art), with font sizes increased to a minimum of 8 pt for all axis labels, legends, and annotations to meet publication standards. Despite these limitations, the concordance of bioinformatic predictions with experimental data across multiple platforms (TCGA, GEO, qRT-PCR, ELISA) substantially strengthens confidence in the biological validity of the identified hub genes.

In conclusion, we have identified a five-gene cell cycle hub (EZH2, CDK1, AURKA, TOP2A, CCNB1) in the TCGA-PRAD turquoise co-expression module whose collective expression characterizes a clinically aggressive and immunosuppressive PCa phenotype. A three-gene machine learning risk model achieves independent prognostic stratification for BCR-free survival, is incorporated into a clinically validated nomogram, and maps onto a quantifiably immunosuppressive microenvironment with therapeutic implications. These findings provide a rationale for prospective validation of the risk score in multi-institutional cohorts and for clinical investigation of combinatorial strategies targeting EZH2, CDK1, and AURKA together with immune checkpoint blockade in high-risk PCa.

## Data Availability

The RNA-seq count data and clinical annotations analyzed in this study are publicly available in the TCGA-PRAD repository through the NCI Genomic Data Commons (GDC) Data Portal. Further inquiries can be directed to the corresponding author.
